# Vestibular-guided visual search

**DOI:** 10.1007/s00221-020-05741-x

**Published:** 2020-02-08

**Authors:** Laura Smith, Annita Gkioka, David Wilkinson

**Affiliations:** School of Psychology, University of Kent, Canterbury, CT2 7NP UK

**Keywords:** Galvanic vestibular stimulation, Multisensory interplay, Visual search, Spatial processing

## Abstract

The amnesic symptoms that accompany vestibular dysfunction point to a functional relationship between the vestibular and visual memory systems. However, little is known about the underpinning cognitive processes. As a starting point, we sought evidence for a type of cross-modal interaction commonly observed between other sensory modalities in which the identification of a target (in this case, visual) is facilitated if earlier coupled to a unique, temporally coincident stimulus from another sensory domain (in this case, vestibular). Participants first performed a visual detection task in which stimuli appeared at random locations within a computerised grid. Unknown to participants, the onset of one particular stimulus was accompanied by a brief, sub-sensory pulse of galvanic vestibular stimulation (GVS). Across two visual search experiments, both old and new targets were identified faster when presented in the grid location at which the GVS-paired visual stimulus had appeared in the earlier detection task. This location advantage appeared to be based on relative rather than absolute spatial co-ordinates since the effect held when the search grid was rotated 90°. Together these findings indicate that when individuals return to a familiar visual scene (here, a 2D grid), visual judgements are facilitated when targets appear at a location previously associated with a unique, task-irrelevant vestibular cue. This novel case of multisensory interplay has broader implications for understanding how vestibular signals inform cognitive processes and helps constrain the growing therapeutic application of GVS.

**Electronic supplementary material:**

The online version of this article (10.1007/s00221-020-05741-x) contains supplementary material, which is available to authorized users.

## Introduction

Deep within the inner ear and enclosed by dense temporal bone lie the vestibular organs, small but complex structures that sense orientation and movement of the head (Highstein 2004). The vestibular system informs our ‘inner GPS’ by telling us which way is up, whether we are moving, and if so in what direction and at what speed. This information is continuously integrated with visual and proprioceptive inputs to stabilise gaze and posture, and also influences higher-level egocentric functions encompassing affective, perceptual and attentional processing, with visual-spatial memory showing an especially strong reliance (Smith 2017).

Animal experiments dating back to the 1960s (i.e., Beritoff 1965) show that disturbance to one or both vestibular labyrinths is associated with a failure to spatially orient and to remember new spatial locations during foraging and navigational tasks (see Smith et al. 2010; Smith and Zheng 2013 for reviews). Although it is difficult to directly attribute these behavioural impairments to hippocampal dysfunction (not least given that there is no known direct vestibular-hippocampal projection), rats that have undergone vestibular deafferentation show altered long-term potentiation induction in the dentate gyrus and allied increases in *N*-Methyl-d-aspartate glutamate receptor density (Truchet et al. 2019). They also show disrupted activity in their hippocampal place cells (Stackman et al. 2002), post-subicular head-direction cells (Yoder and Taube 2009) and entorhinal grid cells (Jacob et al. 2014). Associations between vestibular and memory function are also readily apparent in humans, as demonstrated by the impaired virtual Morris Water Maze performance that follows partial or complete bilateral vestibular loss (Wiener-Vacher et al. 2013), and by the results of standardised clinical assessments which suggest that individuals who present with vestibular dysfunction are prone to visual-spatial memory and navigational error (Bigelow and Agrawal 2015; Jandl et al. 2015; Smith et al. 2018). Functional neuroimaging in neurologically healthy volunteers also shows robust activation of those temporal and parietal areas associated with visual memory during artificial stimulation of the vestibular system via thermal or galvanic current (Dieterich et al. 2003; Fasold et al. 2002; Suzuki et al. 2001).

Although our clinical and biological understanding of how the vestibular system interacts with visual spatial memory has progressed, psychological accounts remain more limited (Hanes et al. 2006). To date, the main proposed psychological mechanism by which vestibular signals influence visual spatial memory is via a generic arousal effect that operates diffusely and indiscriminately across cognition, affecting sensory-motor, affective, language and executive processes (Smith and Zheng 2013). This idea is premised on several lines of biological evidence including observations that (1) the reticular activating formation, which links to the widespread thalamic-cortical system, is strongly innervated by neighbouring vestibular nuclei (Hitier et al. 2014; Hüfner et al. 2007), (2) caloric vestibular stimulation can increase arousal in moderately to severely brain-injured patients who show either lateralised or global disturbances in awareness (Cappa et al. 1987; Vanzan et al. 2016) and (3) fMRI studies show widespread increases in haemodynamic response, most evidently in the contralateral hemisphere, during galvanic and caloric vestibular stimulation (Dieterich et al. 2003). Building on this idea of general hemispheric arousal, Bächtold et al. (2001) showed that the locations of objects can be recalled quicker if left ear cold water irrigation is administered during encoding while, on the other hand, visually presented words can be recalled quicker if the right ear is stimulated. A similar account has been put forward by Wilkinson et al. (2008) who showed that face recognition, a process partly lateralised to right hemisphere, can be enhanced by right but not left GVS.

It is notable that these studies employed experimental paradigms that probed vestibular-visual interactions in an unusual manner, discharging a single, unchanging DC or thermal vestibular waveform for minutes at a time during which multiple visual stimuli were presented. Such a waveform is physiologically implausible because it mirrors a real-world situation in which the head is continually rotating along the same head movement vector (Angelaki and Cullen 2008). Moreover, by presenting multiple visual stimuli during a simulated and prolonged head movement, it is difficult to establish how vestibular signals affect individual stimulus encoding; an important capacity given that individual stimuli typically form the focus of visually-guided action. It is also notable that with the exception of Wilkinson et el. (2008) the above vestibular stimulation studies applied super-sensory waveforms that elicit conscious sensations (e.g. itching/tingling under the GVS electrodes, feelings of vertigo, light-headedness or self-motion) and, accordingly, have elicited central effects that can be attributed to generic attentional arousal rather than activation of latent vestibular origin.

We raise the above queries because many visual events are brief and often accompanied by a unique vestibular signal—at any one moment in time, the movement and position of the head is often different from the last. It is conceivable that visual processes use this unique, coincident information to help enrich or individuate stimulus encoding, as is the case in other cross-modal interactions (Lehmann and Murray 2005). For example, it is now well established that the perception of a visual stimulus is enhanced if temporally coincident with a distinctive sound or tactile stimulus (Driver and Spence 2000; Laurienti et al. 2004; Lehmann and Murray 2005). Other studies indicate that the features of irrelevant, background stimuli can influence later target identification. For example, Chun and Jiang (1998) showed that embedding targets within familiar configurations of distracter stimuli reduced search times, an advantage that they attribute to a contextual cuing effect in which the deployment of visual attention is sensitive to the broader perceptual context in which target stimuli are encoded (see Kristjánsson and Campana 2010). They argue that this context is incidentally learned over time and forms an implicit memory that guides search in subsequent encounters. In the present study, the question arises as to whether the search for a target within a familiar scene is biased towards a stimulus that was previously associated with a distinctive head movement. That is, whether implicit information about head position can shape the broader perceptual context in which the search for individual visual stimuli occurs.

To investigate the above issue, we generated a novel paradigm to determine if vestibular signals can be paired with concurrent visual stimuli in a way that facilitates their later search and identification. Brief pulses of GVS, which act to simulate a natural head movement (Fitzpatrick and Day 2004), were paired with the onset of a unique visual stimulus which later had to be found within a visual search array. The GVS pulses were applied at a sub-sensory level so that no cutaneous sensation or illusory head movement was felt. The GVS pairing process was accomplished during a detection task in which participants viewed an empty grid and pressed a button as soon as either a distractor dot or novel object, known as a ‘fribble’ (see Barry et al. 2014), appeared somewhere within it. When a single, pre-determined target fribble appeared, a brief GVS pulse was discharged. In the subsequent visual search task, participants were shown a picture of a fribble and then asked if it was present amongst other fribbles in the search display. Some of the fribble search targets had been presented in the earlier detection task (i.e. primed), one of which had been paired with the GVS pulse, while others were new (i.e. un-primed). We reasoned that if vestibular signals can facilitate visual target identification, similar to other cross-modal interactions, then identification would be enhanced for stimuli paired with a GVS pulse.

**Fig. 1:**
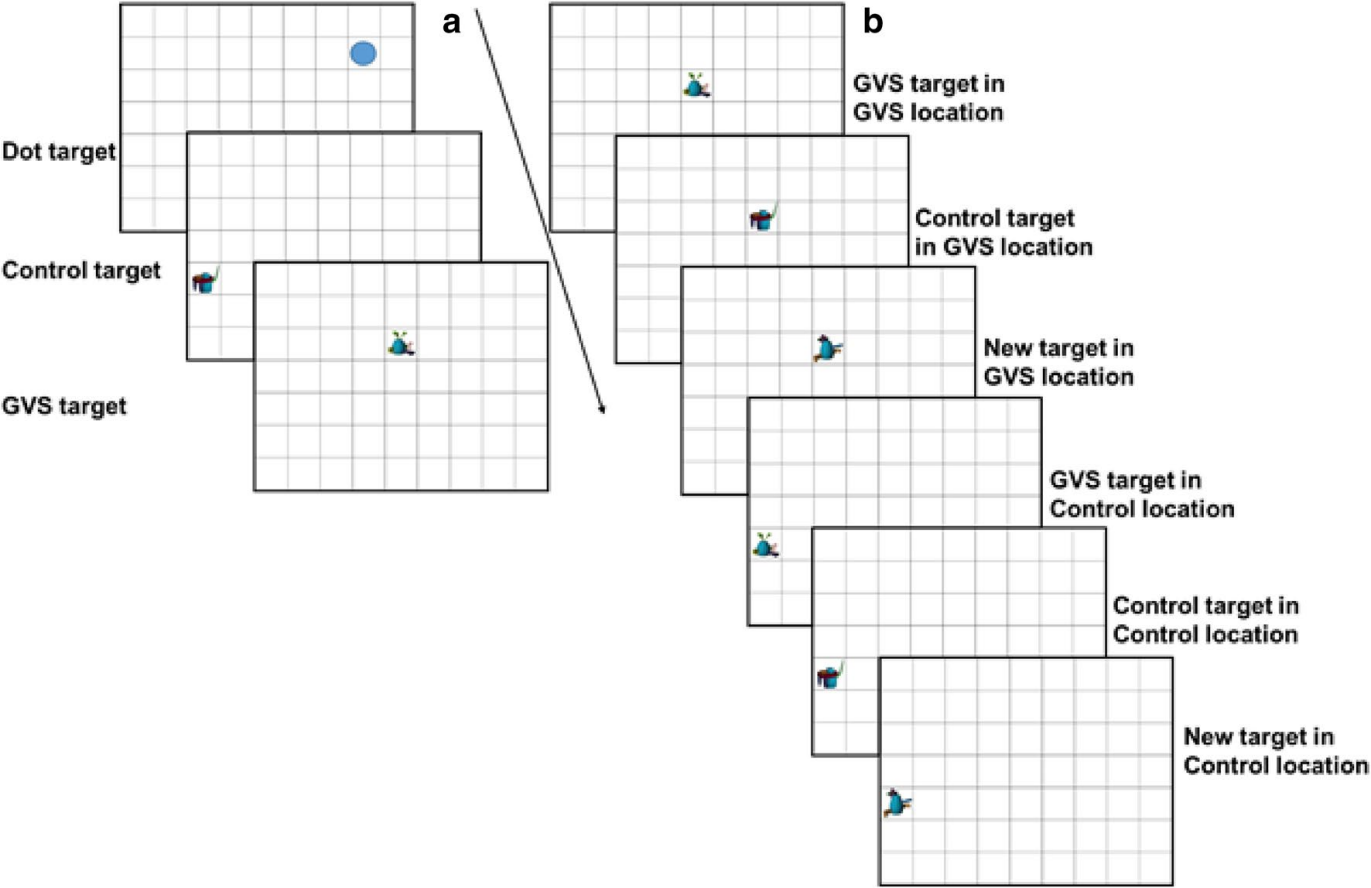
Experimental design. **a** Example fribble and coloured dot stimuli from the detection task, including the GVS-paired stimulus and Control stimuli shown in their primed locations. The identities and locations of the GVS and Control stimuli were counterbalanced across pairs of participants. **b** Example target present trials from the search task. GVS, Control and Newtarget images were shown in the GVS primed spatial location or the Control location from the detection task. The search displays also contained distracter stimuli which are not shown here

To cast light on the source of any enhancement, we also explored whether any benefit was specific to stimulus location and/or identity. In trials in which the GVS-paired fribble appeared, it sometimes appeared in the same grid location as in the earlier detection task. In other trials, the GVS-paired fribble appeared in a new grid location to enable us to specifically test if object identity had been primed. In other trials, a different target—either one seen in the detection task (control) or an entirely new one—appeared in the spatial location at which the GVS fribble had been primed (see Fig. 1). We hypothesised that if GVS primes spatial location then the search for GVS-paired targets should be more efficient when they re-appear at their earlier location compared to when they appear elsewhere. Alternatively, if GVS only primes target identity then no location effect should be found, although search for GVS-paired fribble stimuli should be better overall. Of course, if GVS primes both location and identity then GVS targets should be the easiest to find wherever they appear, but with those appearing at the initial encoding location proving especially easy.

## Experiment 1—Visual search for GVS-Paired visual stimuli

### Materials and methods

#### Participants

Sixty participants completed the protocol in Experiment 1. The sample size was informed by a power calculation indicating that a sample size of *N* = 39 would be required to detect a moderate effect (Cohen’s *f* = 0.3) in a repeated measures ANOVA with an *α* of 0.05 and a *β* of 0.95. Given uncertainty over the true underlying effect size of pairing GVS with a single stimulus and participant compliance, we recruited beyond this number.

All participants were psychology students from the University of Kent. Participants with a self-reported history of vestibular or hearing disorder were excluded in case this disrupted transmission of the GVS signal. The experimental protocol was approved by the School of Psychology Ethics Committee at the University of Kent (School ethics code = 2718) and the study was conducted in line with the 1964 Helsinki Declaration. All subjects gave written informed consent prior to study commencement.

#### Experimental materials

Stimuli for the search task were taken from a pool of novel objects known as ‘fribbles’ (Barry et al. 2014) and resized to 119^2^ pixels. As recommended by Manelis et al. (2011), coloured dots were also presented in the detection task (the colours matched those of the fribble bodies) to discourage participants from dwelling on the identities of stimuli and from developing explicit coding strategies which may have induced an unwanted advantage in the later search task. Stimuli appeared on a 30 × 23 cm grid with individual squares of 124^2^px created with the GNU Image Manipulation Program. All stimuli were shown on a white background projected by a 15 inch display monitor running E-prime® software with a viewing distance of 40 cm.

A padded chin rest held participants’ head position constant to minimise natural vestibular stimulation. Free head movement was permitted during breaks.

#### Design and procedure

Participants completed 13 block repetitions within a single experimental session. Each block comprised a detection task, comprising 31 trials, which was repeated three consecutive times (to increase priming). After a brief 100 s break, participants completed the search task comprising 20 trials (performed just once per block). The experiment lasted 1.5 h and participants were debriefed upon completion.

##### Detection task

Each trial began with an empty grid displayed centrally for 550 ms, after which individual fribbles or dots were then displayed for a maximum of 1000 ms each. Participants were instructed to press the spacebar as quickly as possible when a stimulus appeared within the grid (adapted from Manelis et al. 2011). Each repetition of the detection task included 21 unique fribbles and 10 dots presented in random order. A sub-sensory GVS pulse was discharged to match the onset of one pre-defined target stimulus which remained the same across the experiment. Different participants were assigned different target stimuli. Across participants, the stimulus identities and locations of the GVS and control stimuli were counterbalanced so that if a stimulus was the GVS prime for one participant then it served as the control stimulus for another (see Fig. 1).

##### Search task

Participants were first presented with a single target displayed centrally at the top of the screen for 2000 ms and asked to report, as quickly and as accurately as possible in the forthcoming display, whether it was present or absent. This target object was either an ‘old’ image that the participant had viewed during the detection task, or a ‘new’ image that appeared for the first time. The target then disappeared and after a variable ISI (500–800 ms) a search display of 12 objects appeared. ‘Old’ target objects were either presented in the same grid location at which they were displayed during the detection task or in a different location. In target present trials, one of the ‘old’ objects had been paired with the GVS signal in the previous detection task. Participants clicked on the target object with the mouse cursor if it was present or clicked on a 'Not-present' button above the search display if it was absent. Participants completed 20 search trials (10 absent, 10 present) with trial type order randomised. Four different search arrays were created for each experiment, each comprising different stimulus arrangements.

#### Galvanic vestibular stimulation (GVS)

Participants’ mastoid processes were first exfoliated to reduce impedance, after which 6 cm × 5 cm self-adhesive carbon rubber electrodes were attached and then connected via insulated cables to a neuroConn DC-Stimulator. A boxcar pulse of 0.3 mA bilateral, bipolar direct current (anode left, cathode right) lasting 1000 ms was triggered by E-Prime® and discharged to match the onset of the target stimulus during the detection task. An amplitude of 0.3 mA was applied because previous studies indicate that this is too small to elicit cutaneous or vertiginous sensation and yet reliably activates the vestibular afferents (Day et al. 1997; Séverac et al. 2003). A questionnaire was nevertheless administered at debrief to monitor participants’ perceptions of the electric current (perceived intensity, sensation, onset and frequency). One participant did report a vague cutaneous, mastoid sensation that coincided with a particular stimulus so was subsequently replaced in Experiment 1.

#### Data analysis

Analyses focused on reaction time (RT) and response accuracy from six key target present search task trials to determine whether target identification was enhanced by prior association with GVS (see Fig. 1). We reasoned that if GVS primes spatial location then (1) the search for GVS-paired targets should be more efficient when they re-appear at the same location at which their earlier pairing occurred compared to when they appear elsewhere, and (2) the search for control and new targets should be more efficient when they appear at the primed location than when they appear elsewhere. Conversely, if GVS primes target identity then no location effect should be found across the stimulus types, although search for GVS-paired fribble stimuli should be better overall.

### Results and discussion

#### Data preparation

Although the search task simply required participants to decide whether a target was present, some reported at debrief that they had found it repetitive and laborious. This raised concern that they periodically lost engagement and did not follow the request to respond as quickly and accurately as possible. In line with this, a number of participants performed either at chance or near-chance levels of accuracy. To remove this unwanted influence, only participants who responded correctly to eight or more key target present trials across the 13 search task blocks were included in the analysis below. Consequently, 12 participants were removed from Experiment 1. Importantly, the removal of these participants still enabled the target sample size recommended by the power analysis to be met. Statistical analyses including those participants who performed at or near chance are presented in the supplementary materials section.

RT outliers were removed using a *z*-score correction whereby a grand mean RT was calculated and then subtracted from every individual trial RT, before being divided by a grand standard deviation (*Z* = *Χ* − *μ*/*σ*). Any resulting *z*-scores that were greater than 2.5 standard deviations (and were, therefore, an outlier of less than *p* < 0.001) were removed from the data.

Importantly, these data were removed during data preparation before summary scores for each experimental condition were calculated and before statistical hypothesis testing.

RTs for dots and all fribble stimuli were first compared during the detection task to check for the use of explicit memory strategies. Participants took longer to respond to dot stimuli (*M* = 254 ms) compared to the fribble stimuli (*M* = 250 ms) [*t*(47) = 3.94, *p* < 0.001], suggesting that the identities of fribbles had not received additional processing which might later influence search.

Second, to confirm that primed stimuli from the previous detection task had been committed, albeit maybe inadvertently, to memory—and that potential, therefore, existed for the GVS prime to interact with memorial rather than only perceptual processes—mean correct filtered RTs from the search task were then compared across all old and new targets. The expected priming effect was present [*t*(47) = 8.49, *p* < 0.001] such that old items (*M* = 1558 ms) were responded to more quickly than new items (*M* = 1729 ms).

After completing these data checks a repeated-measures ANOVA with Target (GVS, Control, New), and Location (GVS, Control) as within-subject factors then compared correct, *z*-score filtered RTs and accuracy scores from the target present trials.

#### Reaction time

**Fig. 2:**
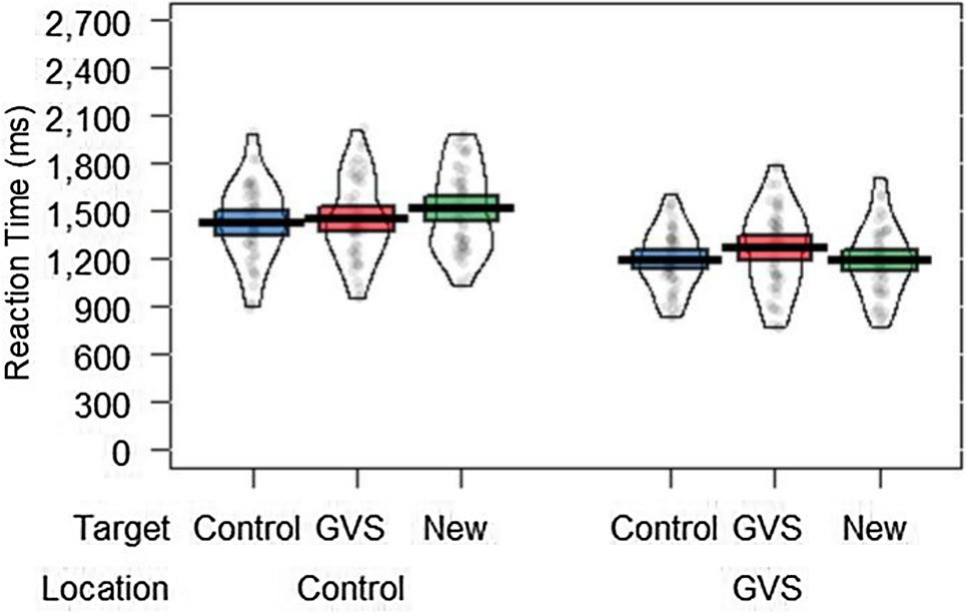
Mean reaction times for the target-present trials in Experiment 1. The bold horizontal line indicates the group mean, the band indicates the 95% confidence intervals, the bean shows the data distribution and the points show the raw data

A significant main effect of Location [*F*(1, 47) = 45.67, *p* < 0.001, *η*_p_^2^ = 0.493] revealed shorter RTs towards targets presented in the GVS location (*M* = 1220 ms) compared to the Control location (*M* = 1465 ms)*.* Descriptive statistics revealed that at least 79% of participants showed this effect of Location. There was no significant main effect of Target (*p* = 0.21). However, the two-way interaction between Target and Location was significant, *F*(2, 94) = 7.40, *p* < 0.05, *η*_p_^2^ = 0.136. Bonferroni-corrected (*α* = 0.05) pairwise comparisons indicated that responses were shorter towards all Targets (GVS, Control, New), but particularly the New Target, when displayed within the GVS location relative to the Control location (see Fig. 2 ; all *t*s < 7.43, all *p*s < 0.001). No post-hoc comparisons involving Target reached significance (all *t*s < 2.24, all *p*s > 0.09).

#### Accuracy

Mean accuracy remained consistently high across all conditions (*M* = 0.93), with no statistical effects reaching significance (all *F*s < 1.38, all *p*s > 0.26).

The results from Experiment 1 indicate that implicitly coupling a visual stimulus to a brief pulse of GVS can speed the rate at which stimuli appearing at that location are later found during a search task. Interestingly, the effect extended to targets that had not appeared there before (i.e. new targets) and even applied to targets that had been initially encoded during the detection task at another location (i.e. control targets). Experiment 2 sought to replicate this effect in a new participant sample with new stimuli, and to further characterise the spatial advantage.

## Experiment 2—Visual search for GVS targets in upright versus rotated orientation

The RT advantage reported in Experiment 1 was specific to the spatial location at which a visual stimulus happened to appear when the GVS signal was discharged. This may imply that the location effect was retinotopic. However, physiological studies show that vestibular signals influence the activity of head direction and place cells in hippocampus (see Hitier et al. 2014 for a review) which are not retinotopic, and instead encode spatial locations relative to other points of reference. This form of relative coding ensures that objects can still be found when their retinotopic coordinates change because the location of the individual or visual set has shifted. In Experiment 2, we, therefore, rotated the search displays 90º to test if the priming effects observed in Experiment 1 were maintained when the relative positions of targets in the priming grid were maintained but their absolute positions were altered. Displays were also presented at their upright orientation to enable a replication of the effects reported in Experiment 1.

### Materials and methods

Experiment 2 was identical to Experiment 1 with the following changes:

#### Participants

Sixty-seven different participants completed Experiment 2. Given the replicatory nature of study, this sample size was partly informed by the above power analysis from Experiment 1. Concern over participant non-compliance again led us to recruit additional participants.

#### Design and procedure

A between subjects manipulation was added so that half of the participants (*N* = 36) viewed the display in the same upright orientation during the detection and search tasks, while the others viewed the display in an upright orientation during the detection task and then in the rotated version (90° to the right) during search (*N* = 31).

To help participants acquire the layout of the rotated condition, four peripheral cues (coloured and shaped differently to the fribbles) were placed at each corner. Effort was made to position targets away from the cues in case they made search especially easy (see Fitting et al. 2009).

### Results and discussion

#### Data preparation

Only participants who responded correctly to eight or more target present trials across the 13 search task blocks were included in the analysis. Consequently, 27 participants were removed from Experiment 2 leaving a sample size of 40 (*N* = 20 upright orientation; *N* = 20 rotated orientation). We should point out that if participants who performed at or near chance are included in the analyses then the main pattern of effects reported below are retained, but the effects in this Experiment are weaker (see supplementary materials).

During the detection task participants’ responses were shorter [*t*(39) = 6.62, *p* =  < 0.001] towards the fribble (*M* = 250 ms) than dot stimuli (*M* = 254 ms), again suggesting that explicit memory processing strategies were not applied to the fribble stimuli. In the search task, the expected old/new priming effect was again present [*t*(39) = 3.10, *p* < 0.05] with old items (*M* = 1648 ms) generating shorter search times than new items (*M* = 1716 ms). A 3 (Target: GVS, Control, New) × 2 (Location: GVS, Control) Repeated Measures ANOVA was then conducted on the mean correct, *z*-score filtered, target present RTs and accuracy scores with Orientation (Upright, Rotated) as a between-subjects factor.

#### Reaction time

**Fig. 3:**
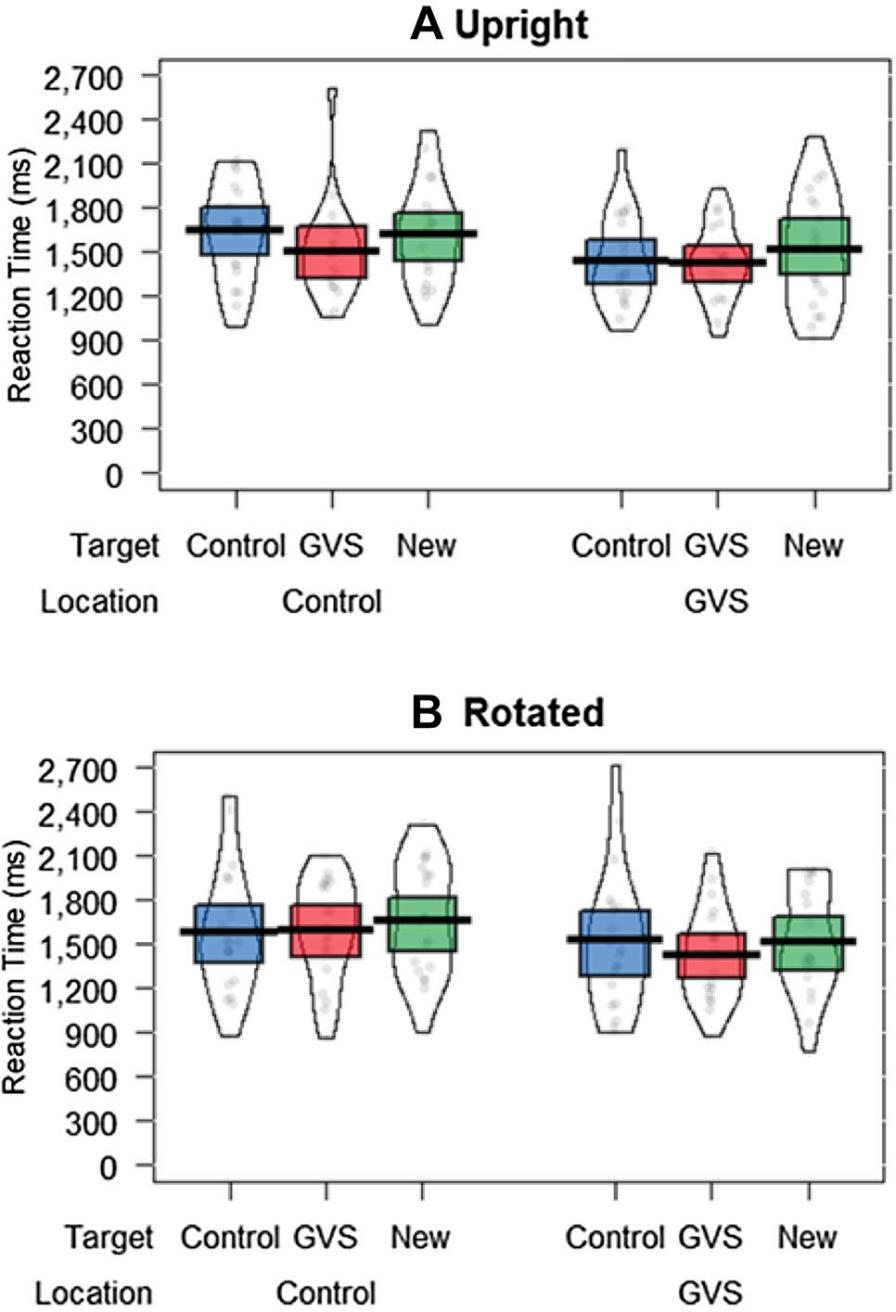
Mean reaction times in the upright (**a**) and rotated (**b**) conditions for the target-present trials in Experiment 2

Once again there was a significant main effect of Location, *F*(1, 38) = 7.72, *p* < 0.01, *η*_p_^2^ = 0.169, associated with shorter responses when targets were presented in the GVS (*M* = 1477.46 ms) rather than the Control (*M* = 1604.12 ms) location. Descriptive statistics revealed that 70% of participants showed this effect of Location (see Fig. 3). Follow-up analyses also confirmed that the effect of Location held in both Orientation conditions. A one-tailed *t *test (motivated by the replicatory nature of the comparison) showed that the Location effect from Experiment 1 was replicated in the upright display [*t*(19) = 1.87, *p* < *0.05*], and a two-tailed *t *test showed the effect also applied to the rotated [*t*(19) = 2.10, *p* < 0.05] display.

There was also a marginal main effect of Target, *F*(2, 76) = 2.70, *p* = 0.07, *η*_p_^2^ = 0.07, which seemed to reflect the finding that RTs were shorter towards the GVS Target (*M* = 1492.38 ms) than the New Target (*M* = 1579.40 ms). The main effect of Orientation and all other interactions failed to reach significance (all *F*s < 2.30, all *p*s > 0.11).

#### Accuracy

As in Experiment 1, average accuracy was consistently high across all conditions (*M* = 0.88). No significant main effects nor any interactions between Target, Location and Orientation were present (all *F*s < 1.04, all *p*s > 0.36).

## General discussion

Physiological and clinical data from individuals with vestibular dysfunction show a close interplay between the vestibular and visual-spatial short-term memory systems (see Bigelow and Agrawal 2015 and Smith 2017 for reviews). However, the psychological processes involved in this interplay have not been established. Previous psychological studies have focused on trying to enhance performance rather than identify underlying mechanisms of action, and partly as a consequence, have discharged prolonged waveforms that are physiologically unnatural. Experimental outcomes have been described with reference to a post-hoc mechanistic account based on hemispheric arousal that does not speak to how visual processes are specifically affected. The aim of the present study was to establish whether brief vestibular signals that more faithfully reflect the time-frame of natural head movements can interact with co-temporaneous visual stimuli in a fine-grained manner, improving the efficiency with which individual visual stimuli can later be found in a multi-object array.

Experiment 1 combined an implicit priming and visual search paradigm to determine whether a visual stimulus that was encoded alongside a sub-sensory, incidental vestibular signal would later be found more quickly than unpaired visual stimuli. As predicted, visual targets were found quicker in a multi-object search array when presented in a location that had been encoded earlier during receipt of GVS. Analysis showed that the priming effect was location rather than identity specific because all stimuli, regardless of whether they were old or new, that appeared in the primed location were found quicker. Experiment 2 replicated this effect in a new group of participants using new search arrays. In addition, the effect was maintained when search displays were rotated so that the absolute coordinates of the primed location changed but the location of the prime relative to other stimuli on the computer screen stayed the same. This result indicates that the GVS signal was priming representations of relative rather than only absolute (or retinotopic) space. More generally, it indicates that when individuals return to a familiar visual scene (i.e. a 2D grid), judgements are facilitated for stimuli that appear at a location that was previously associated with a unique vestibular stimulus.

A feature of the facilitation observed here (and unlike that reported during auditory and tactile cross-modal priming) is that the vestibular stimulus was sub-sensory so could not be easily attributed to overt attentional arousal effects. That is, it did not elicit conscious sensation such as itching, pins and needles or illusory head movement. The implicit nature of this vestibular prime is perhaps unsurprising given that the vestibular system is, unless damaged, a silent sense with no perceptible sensation. We suggest that there are at last two ways in which the prime might have biased later visual search; (1) its unexpected/salient onset sharpened the concurrent encoding of visual stimuli which in turn were stored in memory more effectively, and/or (2) the visual location associated with the vestibular prime was retained within a multisensory representation of the search array enabling it to directly bias future deployments of attention. One can only speculate on the adaptive value of such a bias, but it would seem to make sense to maintain a record of where visual targets are located during salient/unexpected head movement so that sensory responses can be primed to that part of the visual array the next time it is encountered. This might especially be true for head movements associated with a fight or flight response, and for head movements as unexpected as those signalled by the GVS pulse. Such an account fits more broadly with the idea that visual search is not only affected by current search conditions but also by learned associations between target stimuli and the broader perceptual context in which they have been previously encountered (see Chun and Jiang 1998; Kristjánsson and Campana 2010).

The finding that a task-irrelevant but temporally coincident vestibular stimulus can influence subsequent visual search has not been previously shown. Demonstrations of vestibular-visual interactions have instead been mostly restricted to feed-forward multisensory convergence in which inputs from the vestibular and visual senses are combined to reduce perceptual uncertainty about a common egocentric property such as the perception of subjective vertical or self-motion (Angelaki et al. 2009). In the present case, the vestibular inputs inform on judgments (i.e. visual target identification) that logically only apply to the visual modality. As reviewed above, one or two former studies have alluded to such an effect but have applied unnaturally long vestibular stimuli and have not been applied in a way that can show a link between individual head movements and judgements about individual visual stimuli; these studies have instead invoked a mechanism based on non-specific cognitive arousal which tells us little about the specific manner in which vestibular information can guide memory. Somewhat similar facilitation effects have been found within the somatosensory pathway in which co-temporaneous vestibular signals can increase sensitivity to mechanical and electrical stimuli (Ferrè et al. 2011, 2014), but these somatosensory effects were observed during GVS as opposed to afterwards so probed perceptual rather than memorial processes. Nevertheless, these earlier perceptual findings do suggest that the unimodal influence observed here reflects a more general feature of vestibular-visual interplay.

Beyond furthering our understanding of how vestibular signals guide visual search, the current results further justify and constrain the therapeutic application of vestibular stimulation. Symptomatic relief of hemi-inattention and short-term memory loss has been observed following continuous, prolonged (i.e. ~ 20–30 mins) periods of stimulation (Ghahraman et al 2016; Wilkinson et al. 2010, 2014; 2019). The present data raise the possibility that much briefer, stimulus-locked periods of stimulation may also bring benefit. In the case of hemi-inattention (and perhaps also hemianopia), the question arises as to whether GVS can be used to drive attention towards neglected space by endogenously marking a single, peripheral, field location. The beneficial effect of spatial cuing in hemi-inattention is long-established but this is typically achieved by verbal prompts or by attaching salient, visual markers to physical objects located in neglected space (Bailey et al. 2002) rather than by implicitly priming spatial processes in the manner described here. A related, although admittedly more speculative, question is whether GVS could be used to prime key spatial locations (such as one’s home) within the relevant topographic memories of those whose memory loss compromises their navigational ability and direction-finding. In support of this idea, previous research has shown that low amplitude GVS can speed stimulus discriminations that rely on visual imagery (Wilkinson et al. 2008), although it should be highlighted that this study did not investigate the spatial specificity of the GVS advantage. Nevertheless, we encourage others to now consider how brief, co-temporaneous pulses of GVS could be used to enhance the process of stimulus individuation in people with perceptual or memorial loss.

The insights gained from the current study are clouded by at least several ambiguities. Recall that the GVS prime was applied in the detection task at the same time that a to-be-detected visual stimulus was presented and a response button had to be pressed. One is inclined to believe that participants were attending the visual stimulus during this period and that, accordingly, the prime interacted with visual attentional processes. However, it is also possible that the prime interacted either at an earlier pre-attentive stage of stimulus registration or at the level of response selection/execution. It is also possible that GVS-induced ocular torsion which provided additional sensory-motor feedback during encoding. Such possibilities could perhaps be unpicked in future studies using eye-tracking. A second ambiguity is whether the current findings only occur when GVS, as opposed to a real head movement, is used to generate vestibular stimuli. Although GVS is widely taken to simulate a natural head movement, the movement is incongruent with head position information conveyed by the visual and proprioceptive senses (Palla and Lenggenhager 2014). This mismatch could amplify the salience of the vestibular signal and raises the question as to whether more predictable and natural head movements elicit the same magnitude of effect. Individual differences in sensory preference could also interact with the salience of the vestibular signal. Some individuals place particular reliance on vestibular estimates of direction and distance during spatial tasks, while others are more reliant on visual cues such as landmarks and optic flow (Hüfner et al. 2011). Simple measures of sensory preference such as the Visual Vertigo Scale (Dannenbaum et al. 2011) or Rod and Frame Test (Witkin and Asch 1948) might help explain individual response variability in tasks that seek to moderate visual response via vestibular cues.

## Conclusions

To summarise, we show for the first time that the vestibular system can facilitate subsequent visual judgements via a form of multisensory modulation that hitherto has not been observed within the vestibular system. Previous studies of vestibular-visual interaction have mostly focused on multisensory convergence, while those few that have focused on visual-spatial memory have utilised experimental paradigms unable to capture such forms of interplay. Future studies will need to explore other conditions in which vestibular and visual stimuli can be coupled to facilitate visual performance in both clinical and non-clinical populations.

## Electronic supplementary material

Below is the link to the electronic supplementary material.

Supplementary file1 (DOCX 14 kb)

## Data Availability

Data available on the Open Science Framework: https://osf.io/fe3mk/?view_only=6f28b478aa154bfcb77cce5228a1ee51

## References

[CR1] Angelaki DE, Cullen KE (2008). Vestibular system: the many facets of a multimodal sense. Annu Rev Neurosci.

[CR2] Angelaki DE, Klier EM, Snyder LH (2009). A vestibular sensation: probabilistic approaches to spatial perception. Neuron.

[CR3] Bächtold D, Baumann T, Sandor PS, Kritos M, Regard M, Brugger P (2001). Spatial-and verbal-memory improvement by cold-water caloric stimulation in healthy subjects. Exp Brain Res.

[CR4] Barry TJ, Griffith JW, De Rossi S, Hermans D (2014). Meet the Fribbles: novel stimuli for use within behavioural research. Front Psychol.

[CR5] Bailey MJ, Riddoch MJ, Crome P (2002). Treatment of visual neglect in elderly patients with stroke: a single-subject series using either a scanning and cueing strategy or a left-limb activation strategy. Phys Ther.

[CR6] Bigelow RT, Agrawal Y (2015). Vestibular involvement in cognition: visuospatial ability, attention, executive function, and memory. J Vestib Res.

[CR7] Beritoff JS (1965). Neural mechanisms of higher vertebrates.

[CR8] Brandt T, Schautzer F, Hamilton DA, Brüning R, Markowitsch HJ, Kalla R, Darlington C, Smith P, Strupp M (2005). Vestibular loss causes hippocampal atrophy and impaired spatial memory in humans. Brain.

[CR9] Cappa S, Sterzi R, Vallar G, Bisiach E (1987). Remission of hemineglect and anosognosia during vestibular stimulation. Neuropsychologia.

[CR10] Chun M, Jiang Y (1998). Contextual cueing: implicit learning and memory of visual context guides spatial attention. Cogn Psychol.

[CR11] Dannenbaum E, Chilingaryan G, Fung J (2011). Visual vertigo analogue scale: an assessment questionnaire for visual vertigo. J Vestib Res.

[CR12] Day B, Séverac Cauquil A, Bartolomei L, Pastor M, Lyon IN (1997). Human body-segment tilts by galvanic stimulation: a vestibularly driven balance protection mechanism. J Physiol.

[CR13] Dieterich M, Bense S, Lutz S, Drzezga A, Stephan T, Bartenstein P, Brandt T (2003). Dominance for vestibular cortical function in the non-dominant hemisphere. Cereb Cortex.

[CR14] Driver J, Spence C (2000). Multisensory perception: beyond modularity and convergence. Curr Biol.

[CR15] Fasold O, von Brevern M, Kuhberg M, Ploner CJ, Villringer A, Lempert T, Wenzel R (2002). Human vestibular cortex as identified with caloric stimulation in functional magnetic resonance imaging. Neuroimage.

[CR16] Ferrè ER, Bottini G, Haggard P (2011). Vestibular modulation of somatosensory perception. Eur J Neurosci.

[CR17] Ferrè ER, Kaliuzhna M, Herbelin B, Haggard P, Blanke O (2014). Vestibularsomatosensory interactions: effects of passive whole-body rotation on somatosensory detection. PLoS ONE.

[CR18] Fitting S, Wedell DH, Allen GL (2009). Cue effects on memory for location when navigating spatial displays. Cognit Sci.

[CR19] Fitzpatrick RC, Day BL (2004). Probing the human vestibular system with galvanic stimulation. J Appl Physiol.

[CR20] Ghahraman MA, Zahmatkesh M, Pourbakht A, Seifi B, Jalaie S, Adeli S, Niknami Z (2016). Noisy galvanic vestibular stimulation enhances spatial memory in cognitive impairment-induced by intracerebroventricular-streptozotocin administration. Physiol Behav.

[CR21] Hanes DA, McCollum G (2006) Cognitive-vestibular interactions: a review of patient difficulties and possible mechanisms. J Vestib Res16(3): 75–91. Retrieved from https://content.iospress.com/journals/journal-of-vestibularresearch/26/5-6 17312336

[CR22] Highstein SM, Highstein SM, Fay RR, Popper AN (2004). Anatomy and physiology of the central and peripheral vestibular system: overview. The vestibular system.

[CR23] Hitier M, Besnard S, Smith PF (2014) Vestibular pathways involved in cognition. Front Integr Neurosci. 10.3389/fnint.2014.00059 PMC4107830 25100954

[CR24] Hüfner K, Hamilton DA, Kalla R, Stephan T, Glasauer S, Ma J, Strupp M (2007). Spatial memory and hippocampal volume in humans with unilateral vestibular deafferentation. Hippocampus.

[CR25] Hüfner K, Strupp M, Smith P, Brandt T, Jahn K (2011). Spatial separation of visual and vestibular processing in the human hippocampal formation. Ann NY Acad Sci.

[CR26] Jandl NM, Sprenger A, Wojak JF, Göttlich M, Münte TF, Krämer UM, Helmchen C (2015). Dissociable cerebellar activity during spatial navigation and visual memory in bilateral vestibular failure. Neuroscience.

[CR27] Jacob PY, Poucet B, Liberge M, Save E, Sargolini F (2014). Vestibular control of entorhinal cortex activity in spatial navigation. Front Integr Neurosci.

[CR28] Kremmyda O, Hüfner K, Flanagin VL, Hamilton DA, Linn J, Strupp M, Brandt T (2016). Beyond dizziness: Virtual navigation, spatial anxiety and hippocampal volume in bilateral vestibulopathy. Front Hum Neurosci.

[CR29] Kristjánsson Á, Campana G (2010). Where perception meets memory: a review of repetition priming in visual search tasks. Atten Percep Psychophys.

[CR30] Laurienti PJ, Kraft RA, Maldjian JA, Burdette JH, Wallace MT (2004). Semantic congruence is a critical factor in multisensory behavioral performance. Exp Brain Res.

[CR31] Lehmann S, Murray MM (2005). The role of multisensory memories in unisensory object discrimination. Cognit Brain Res.

[CR32] Manelis A, Hanson C, Hanson S (2011). Implicit memory for object locations depends on reactivation of encoding-related brain regions. Hum Brain Mapp.

[CR33] Palla A, Lenggenhager B (2014). Ways to investigate vestibular contributions to cognitive processes. Front Integr Neurosci.

[CR34] Schautzer F, Hamilton D, Kalla R, Strupp M, Brandt T (2003). Spatial memory deficits in patients with chronic bilateral vestibular failure. Ann NY Acad Sci.

[CR35] Séverac A, Faldon M, Popov K, Day B, Bronstein A (2003). Short-latency eye movement evoked by near-threshold galvanic vestibular stimulation. Exp Brain Res.

[CR36] Smith L, Wilkinson D, Bodani M, Bicknell R, Surenthiran SS (2018). Short-term memory impairment in vestibular patients can arise independently of psychiatric impairment, fatigue, and sleeplessness. J Neuropsychol.

[CR37] Smith PF (2017). The vestibular system and cognition. Curr Opin Neurol.

[CR38] Smith PF, Darlington C, Zheng Y (2010). Move it or lose it—is stimulation of the vestibular system necessary for normal spatial memory?. Hippocampus.

[CR39] Smith PF, Zheng Y (2013). From ear to uncertainty: vestibular contributions to cognitive function. Front Integr Neurosci.

[CR40] Stackman RW, Clark AS, Taube JS (2002). Hippocampal spatial representations require vestibular input. Hippocampus.

[CR41] Suzuki M, Kitano H, Ito R, Kitanishi T, Yazawa Y, Ogawa T, Kitajima K (2001). Cortical and subcortical vestibular response to caloric stimulation detected by functional magnetic resonance imaging. Cognit Brain Res.

[CR42] Truchet B, Benoit A, Chaillan F, Smith PF, Philoxene B, Guillamin M, Poucet B, Coquerel A, Besnard S (2019). Hippocampal LTP modulation and glutamatergic receptors following vestibular loss. Brain Struct Funct.

[CR43] Vanzan S, Wilkinson D, Ferguson H, Pullicino P, Sakel M (2016). Behavioural improvement in a minimally conscious state after caloric vestibular stimulation: evidence from two single case studies. Clin Rehabilit.

[CR44] Wiener-Vacher SR, Hamilton DA, Wiener SI (2013). Vestibular activity and cognitive development in children: perspectives. Front Integr Neurosci.

[CR45] Wilkinson D, Nicholls S, Pattenden C, Kilduff P, Milberg W (2008). Galvanic vestibular stimulation speeds visual memory recall. Exp Brain Res.

[CR46] Wilkinson D, Podlewska A, Banducci S, Pellat-Higgins T, Bodani M, Sakel M, Smith L, LeWitt P, Ade K (2019). Caloric vestibular stimulation for the management of motor and non-motor symptoms in parkinson's disease. Parkinsonism Relat Dis.

[CR47] Wilkinson D, Zubko O, DeGutis J, Milberg W, Potter J (2010). Improvement of a figure copying deficit during sub-sensory galvanic vestibular stimulation. J Neuropsychol.

[CR48] Wilkinson D, Zubko O, Sakel M, Coulton S, Higgins T, Pullicino P (2014). Galvanic vestibular stimulation in hemi-spatial neglect. Front in Integr Neurosci.

[CR49] Witkin HA, Asch SE (1948). Studies in space perception. III. Perception of the upright in the absence of a visual field'. J Exp Psychol.

[CR50] Yoder RM, Taube JS (2009). Head direction cell activity in mice: robust directional signal depends on intact otolith organs. J Neurosci.

