# Supplementary material for: Vestibular-guided visual search
Source: Exp Brain Res. 2020 Feb 8;238(3):689–98. doi: 10.1007/s00221-020-05741-x (PMC7080682; doi:10.1007/s00221-020-05741-x)
Supplement: Supplementary file 1 — Supplementary file1 (DOCX 14 kb) [file 221_2020_5741_MOESM1_ESM.docx]

Supplementary Materials

*Table S.1*

Reaction Time Analyses Including Those Participants who performed Below Chance in

Experiments 1 and 2.

|  | **ANOVA** | | | |
| --- | --- | --- | --- | --- |
|  | ***df*** | ***F*** | ***P*** | ***ηp²*** |
| *Experiment 1_all participants*  Location  Target  Location*Target | 1, 59 2, 118 2, 118 | 48.61  3.54  8.18 | <0.001**  0.04  <0.001** | .45  .06  .12 |
| *Experiment 2_all participants*  Location^1^  Target Orientation  Location*Target Location*Orientation Target*Orientation Location*Target*Orientation | 1, 63  2, 126  1, 63 2, 126  1, 63 2, 126 2, 126 | 4.99  2.29 0.52  0.24 0.14 0.01 2.85 | 0.03*  0.11  0.47  0.79 0.71 0.99 0.06 | .07  .04  .01 <.01 <.01 <.01  .04 |

**Note.* Significant at *0.05, **<0.01.
Degrees of freedom automatically adjusted following deletion of cases due to missing data.

^1^Follow-up t-tests of Location within the upright [*t*(35)= 1.30, *p*= .20; GVS *M*= 1608.60ms Control *M*= 1686.69ms] and rotated displays [*t*(30)= 2.23, *p*<.05; GVS *M*= 1640.79ms Control *M*= 1785.35ms].
